# Accessing Suicide-Related Information on the Internet: A Retrospective Observational Study of Search Behavior

**DOI:** 10.2196/jmir.2181

**Published:** 2013-01-11

**Authors:** Paul Wai-Ching Wong, King-Wa Fu, Rickey Sai-Pong Yau, Helen Hei-Man Ma, Yik-Wa Law, Shu-Sen Chang, Paul Siu-Fai Yip

**Affiliations:** ^1^Department of Social Work and Social AdministrationFaculty of Social SciencesThe University of Hong KongPokfulamChina (Hong Kong); ^2^Centre for Suicide Research and PreventionFaculty of Social SciencesThe University of Hong KongHong Kong SARChina (Hong Kong); ^3^Journalism and Media Studies CentreThe University of Hong KongHong Kong SARChina (Hong Kong)

**Keywords:** Internet search, Pagerank, Suicide information, Information seeking, Search behavior, Information retrieval

## Abstract

**Background:**

The Internet’s potential impact on suicide is of major public health interest as easy online access to pro-suicide information or specific suicide methods may increase suicide risk among vulnerable Internet users. Little is known, however, about users’ actual searching and browsing behaviors of online suicide-related information.

**Objective:**

To investigate what webpages people actually clicked on after searching with suicide-related queries on a search engine and to examine what queries people used to get access to pro-suicide websites.

**Methods:**

A retrospective observational study was done. We used a web search dataset released by America Online (AOL). The dataset was randomly sampled from all AOL subscribers’ web queries between March and May 2006 and generated by 657,000 service subscribers.

**Results:**

We found 5526 search queries (0.026%, 5526/21,000,000) that included the keyword "suicide". The 5526 search queries included 1586 different search terms and were generated by 1625 unique subscribers (0.25%, 1625/657,000). Of these queries, 61.38% (3392/5526) were followed by users clicking on a search result. Of these 3392 queries, 1344 (39.62%) webpages were clicked on by 930 unique users but only 1314 of those webpages were accessible during the study period. Each clicked-through webpage was classified into 11 categories. The categories of the most visited webpages were: entertainment (30.13%; 396/1314), scientific information (18.31%; 240/1314), and community resources (14.53%; 191/1314). Among the 1314 accessed webpages, we could identify only two pro-suicide websites. We found that the search terms used to access these sites included “commiting suicide with a gas oven”, “hairless goat”, “pictures of murder by strangulation”, and “photo of a severe burn”. A limitation of our study is that the database may be dated and confined to mainly English webpages.

**Conclusions:**

Searching or browsing suicide-related or pro-suicide webpages was uncommon, although a small group of users did access websites that contain detailed suicide method information.

## Introduction

Internet usage is growing exponentially with one third of the world population having Internet access as of 2011, of which 45% were users below the age of 25. The number of Internet users from developing countries has increased from 44% in 2006 to 62% in 2011 [1]. The proliferation and reach of the Internet and its impact on suicide and self-harm behaviors has recently come to the attention of the general public, especially to parents of children and young individuals, suicide prevention professionals, and governments [2]. There is little evidence, however, to either support or refute the notion that the use of the Internet is intrinsically damaging [3].

Infodemiology is a new area of research described as “the science of distribution and determinants of information in an electronic medium,” [4] specifically, the Internet, to inform public health and policy [5]. Recent applications of this methodology include “infoveillance,” the tracking and surveillance of online data for the purpose of predicting influenza outbreaks [6] and the online spread of smoking cessation awareness [7], as well as investigating public search trends in preventing avian flu [8]. The real-time nature of this methodology means that results are timely and may have significant impact in guiding policy making.

In the area of suicide prevention, Dobson [9] has estimated that more than 100,000 suicide-related websites were available on the Internet but due to many search engines’ conservative policies regarding the exposure or sharing of corporate data, there are no reliable data on the prevalence of websites with suicide-related content [10]. The major concerns about the potential role of Internet on suicidal behavior are: 1) the Internet as a major source of readily accessible information on suicide methods and suicidal communication, 2) access to suicide-related information and discussions online by vulnerable, impressionable individuals (eg, the distressed, stressed, and depressed) [11], and young people [12,13] contributes to the increased risk of suicide attempts and completion for them as ways to solve problems or to regulate negative emotions [14,15], and 3) the Internet as an efficient and communal medium for people to meet and arrange suicide pacts [16-18]. Due to the interactive nature of the Internet, Becker and Schmidt [19] argued that the Internet might have even more impact on copycat suicides than print media, eg, the Sorrows of Young Werther. This is the influential book from which the term “Werther effect” arose, which describes the effect of a widely publicized suicide causing emulated suicides.

Search engines on the Internet are often assumed to be an efficient, if not the preferred, gateway for access to suicide-related information [2,20]. A handful of infodemiological studies have been conducted to examine the types of information suicidal individuals might find on the Internet through search engines [15,21-23]. A similar “supply-based” research methodology [4] was adopted in these studies where various self-chosen suicide-related keywords (eg, “suicide”, “suicide methods”, “ways to commit suicide”, or “how to kill yourself”) were entered into several popular search engines, ie, Google, Yahoo!, and the search results were classified into pro-suicide, anti-suicide, or neutral websites. Around 11%, 25%, and 5% of the search result websites were categorized as pro-suicide in the United States, the United Kingdom, and China, respectively [21-23]. Based on these findings, the researchers advocated a number of potential suicide prevention initiatives: 1) Internet service providers and website hosting companies should do more to remove pro-suicide websites [23], 2) Internet service providers may consider strategies to increase the likelihood that vulnerable individuals access helpful rather than potentially harmful websites in times of crisis [21], and 3) enhance access to anti-suicide websites [20] in order to minimize the risks and maximize the benefits of the effects that search engines may have on suicide and suicide prevention. Indeed, a number of national-level policies or initiatives have been implemented in Australia (ie, making the promotion of suicide on the Internet illegal), and in Japan and Korea (ie, specific pro-suicide websites blocked by Internet service providers) [21].

Although prior research has shown that pro-suicide information on methods of suicide are easily accessible through search engines, the findings invited the criticism that the keywords and phrases used were pre-selected by researchers and thus had poor generalizability since the studies were not grounded in the naturalistic search behavior of non-researchers, web-savvy, or suicidal people [24]. Anecdotal evidence has been reported in the literature showing that people who have engaged in attempted suicides had searched for specific ways of committing suicide through the Internet, eg, [25]. There is no empirical data, however, on the general public’s actual web searching behavior for suicide-related information through search engines. This information is needed to generate empirically informed suicide research and prevention strategies on the Internet. The current infodemiological study aims to address this research gap by using a demand-based method [4] investigating the actual searching behavior for suicide-related information on search engines using a publicly accessible dataset released by the America Online (AOL). We examined the search queries people used to access pro-suicide websites through AOL’s search engine. Two research questions regarding suicide and the Internet were examined: 1) What websites do people access to when they search using queries containing “suicide”?, and 2) What search queries (both suicide and non-suicide included) do people use that result in access to pro-suicide websites?

## Methods

### Design

A web search dataset released by the AOL is publicly accessible for data analysis (see similar research in [26]). This dataset is currently the largest publicly accessible search query log on the Internet and represented approximately 1.5% of the total number of search queries conducted through the AOL search engine, which had a 6.7% share of the overall search market in 2006 [27]. The dataset was a random sampling from all AOL subscribers’ search queries between March and May 2006 and consisted of roughly 21 million instances of such queries, collected from roughly 657,000 AOL subscribers [28].

Each entry contained an anonymous service subscriber identity code (ID), time of submission, the rank position, and domain of the clicked-through webpage. A single search query represents one individual click on the “search” button as initiated by a user, regardless of whether the user clicked on any of the webpages in the results page(s). A unique set of one or more keywords used in a search query constitutes the search term(s). A click-through is where a webpage in the returned search results was accessed by an individual click following a single instance of a search query and consequently that webpage is referred to as a clicked-through webpage. The number of click-throughs can also be referred to as traffic.

In response to concerns about privacy, additional measures were taken to replace all possible personal identifiable information from the dataset before undertaking analysis. Each unique subscriber was assigned a random identifier number. The Human Research Ethics Committee for Nonclinical Faculties of the University of Hong Kong approved the ethical aspect of the study protocol.

### Data Coding

To address our first research question, any search query containing the keyword *suicide* in the search term(s) was retrieved from the dataset, and the contents of all clicked-through webpages following these search queries were then content analyzed to derive categories for analysis. Researchers PW and RY independently categorized all of the identified webpages, and each created an initial coding frame. The two coding frames were then compared and refined to develop a consensus coding frame. All of the clicked-through webpages were subsequently individually re-categorized by researchers PW and RY to form the final coding frame. Using the final coding frame, coding disagreements between PW and RY that could not be resolved were referred to the third researcher KWF, who analyzed those blinded to the codes assigned by researchers PW and RY. In most cases, this approach resolved the disagreements but when a decision still could not be made, researchers PW, RY, and KWF jointly visited the clicked-through webpage and discussed the rationale for their coding until agreement was reached.

To address our second research question, web domains from the webpages in the above 11 categories that appeared to encourage suicide or promote the individual right of dying by suicide regardless of medical grounds [23] were identified and content analyzed, and all the search terms used to access these pro-suicide web domains, including ones that did not contain the keyword suicide and additionally the ones found through the first stage of data coding, were identified and examined.

## Results

### Categories of Search Results

We found 5526 search queries (0.026%, 5526/21,000,000) that included the keyword suicide. The 5526 search queries were generated by 1586 unique search terms and 1625 unique users (0.25%, 1625/657,000). The three most frequently used search terms were “suicide” (9.79%, 541/5526), “suicide girls” (5.34%, 295/5526), and “how to commit suicide” (2.17%, 120/5526). Of the 5526 search queries, 3392 (61.38% of 5526) clicked through to a resulting webpage.

Of 3392 search queries with a click-through, 1344 unique webpages were clicked through by 930 unique users using 908 unique search terms. Of the 1344 webpages, 30 webpages were found to be inaccessible during the study period (October to November 2011) and returned error messages such as “page could not be found”, “forbidden access”, or “unable to display page” resulting in 1314 accessible clicked-through webpages. The three most frequently used search terms were also “suicide” (7.61%, 257/3392), “suicide girls” (4.95%, 168/3392), and “how to commit suicide” (2.74%, 93/3392).

Each clicked-through webpage was classified into 11 categories:

1. Entertainment (eg, writing, pictures, music about suicide): Webpages of original writing, pictures, music, or other content that may contain the keyword “suicide” for the purpose of amusement but not necessarily related to the conscious act of killing oneself.
2. Scientific Information: Webpages containing general educational materials, suicide statistics, and research studies.
3. Community Resources: Peer support or non-governmental organization webpages related to suicide prevention.
4. Specific Case/Incident: Webpages related to specific cases of highly publicized suicides.
5. Specific Means: Webpages that explicitly provide details on the methods of suicide, except drug overdose (see below).
6. Overdose and Suicide: There was a noticeable number of webpages that specifically addressed drug overdose or drug-related suicide information, and hence, a separate category from the Specific Means category was created.
7. Religion and Suicide: Webpages with information on suicide from a religious perspective.
8. Physician-Assisted Suicide: Webpages that contained information on euthanasia and physician-assisted suicide.
9. Discussion Forum: Any webpages with an interface primarily for discussion between users.
10. Families and Friends of Suicide/Attempt: Webpages with information about funeral arrangements and resources for family/friends of suicides.
11. Afterlife: Webpages with information and opinions related to “life after death” from suicide as well as life after surviving a suicide attempt. This category was singled out from the “religion and suicide” category because users used search terms that specifically included “afterlife”, and these searches may or may not involve religiosity issues.

Among the 1314 webpages, the proportions of the webpage categories are as follows: entertainment (writings, pictures, music about suicide) (30.14%, 396/1314), scientific information (18.26%, 240/1314), community resources (14.54%, 191/1314), specific case/incident (11.04%, 145/1314), specific means (10.65%, 140/1314), overdose and suicide (4.72%, 62/1314), religion and suicide (4.11%, 54/1314), physician-assisted suicide (2.51%, 33/1314), discussion forum (2.28%, 30/1314), families and friends of suicide/attempt (1.60%, 21/1314), and afterlife (0.91%, 12/1314).

From the search queries, 78.01% of the traffic (2646/3392) went to a webpage link belonging to first page of the search results, 9.17% (311/3392) of the traffic went to the second page of search results, and 10.23% (347/3392) went to the third to fifth page of search results, while only 2.59% (88/3392) trickled to the sixth page or onwards in the search results. These results are generally consistent with current search engine data where links that are higher in the search engine results page, especially on the first page of the search results, tend to get majority of the traffic [29,30].

### Clicked-Through Webpages and Search Terms Containing the Keyword “Suicide”

Table 1 shows the three most commonly used search terms for each webpage category, their usage frequencies, and the number of unique users for each search term. For webpages in the entertainment category, for example, the most commonly used search term that resulted in a click-through to a webpage was “suicide girls”, which was used 247 times (number of queries = 247) by 23 unique users.

**Table 1:** Top three search terms and their search frequencies in each category.

Category	Search term	Frequency	No. of unique users
1) Entertainment (eg, writings, pictures, and music about suicide)	suicide girls	247	23
	suicidegirls	90	11
	suicidegirls.com	46	8
	virgin suicides	46	2
2) Scientific information	suicide	69	49
	free essays on adolescent depression and suicide risks	28	1
	adolescent depression and suicide risks	25	1
3) Community resources	suicide	130	89
	suicide help	29	12
	suicide prevention	16	12
	teen suicide	16	11
4) Specific case/incident	famous suicide 1970 richard cunningham	28	1
	vachel lindsay suicide	16	1
	christopher lee anderson suicide	15	1
5) Specific means	how to commit suicide	70	21
	ways to commit suicide	36	8
	suicide	24	18
6) Overdose and suicide	how to commit suicide	17	13
	cymbalta and suicide	14	1
	suicide by overdose of kadian	8	1
	suicide by overdosing	8	1
7) Religion and suicide	how to commit suicide	13	10
	sermons preach for person who committed suicide	13	1
	suicide in the bible	11	3
8) Physician-assisted suicide	assisted suicide	46	19
	physician assisted suicide	13	8
	should america allow suicide euthanasia	8	1
9) Discussion forum	how to commit suicide	11	10
	Xxsuicide’s xanga site	10	1
	suicideroadmap - myspace blog	9	8
10) Families and friends of suicide/attempt	suicide memorials	17	1
	survivors of suicide	8	3
	suicidememorialwall.com	4	1
11) Afterlife	suicide and the after life	9	1
	survivors of suicide	5	1
	after life suicide	4	1

When suicide was used as a single keyword search term (number of search queries = 256; 19.48%; 256/1314), users mostly clicked through to webpages that belong to the “community resources” (130 search queries), “scientific information” (69 search queries), and “specific means” categories (24 search queries). In contrast, when the search term “how to commit suicide” was used, the users most commonly clicked through to webpages in the “specific means” (70 queries), “overdose and suicide” (17 queries), and “religion and suicide” categories (13 queries).

The search terms most commonly used to access webpages in the “entertainment” category included the keywords in the names of a specific adult website and a novel cum movie. The “specific case/incident” category top search terms involved the keywords in the names of two famous individuals and a teenager who died from suicide; of note, each of these three search queries were contributed by one single user. Webpages in the “specific means” category were most commonly clicked through when users used the search terms “how to commit suicide”, “ways to commit suicide”, and “suicide”.

**Figure 1:**
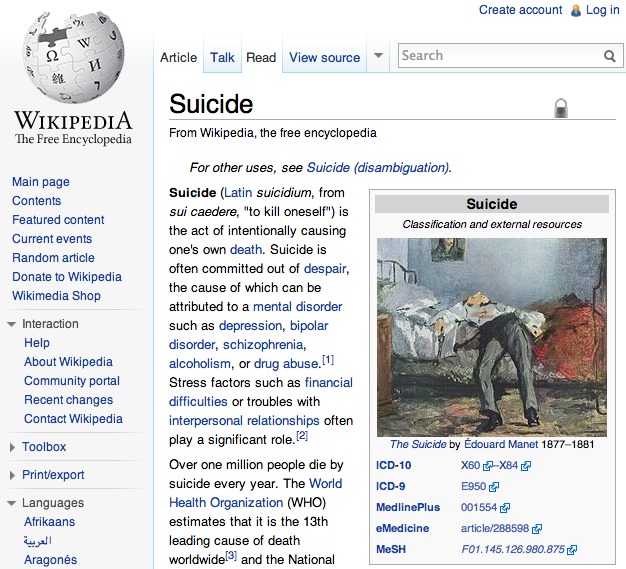
Suicide page on Wikipedia.

Table 2 shows the top three most clicked webpages of each category. The webpages with more than 40 click-throughs during the data collection period included “http://www.suicidegirls.com” (entertainment), “http://suicidegirls.com” (entertainment), “http://metanoia.org” (community resource), “http://www.satanservice.org” (specific means), and “http://www.cdc.gov” (scientific information). In the entertainment category, the two most clicked webpages led to the same web domain, which was an adult website. Wikipedia, an online information resource (Figure 1), followed by an automobile parts branded e-store (“http://www.suicidedoors.com”) were also in the most clicked webpages for the entertainment category. For the specific case/incident category, Wikipedia came up on top again with the most click-throughs, in addition to a news webpage and a social networking webpage.

**Table 2:** Top three most clicked-through webpages of each category, their click-through frequencies, and the number of unique users.

Category	Webpage	Frequency	No. of unique users
1) Entertainment (writings, pictures, music, about suicide)	http://www.suicidegirls.com	153	76
	http://suicidegirls.com	78	59
	http://en.wikipedia.org	21	18
	http://www.suicidedoors.com	21	16
2) Scientific information	http://www.cdc.gov	43	35
	http://www.psycom.net	25	23
	http://www.ncbi.nlm.nih.gov	15	13
3) Community resources	http://www.metanoia.org	86	81
	http://kidshealth.org	20	17
	http://www.afsp.org	16	14
	http://www.focusas.com	16	10
4) Specific case/incident	http://en.wikipedia.org	18	13
	http://news.bbc.co.uk	7	5
	http://profile.myspace.com	7	6
5) Specific means	http://www.satanservice.org	49	39
	http://www.mouchette.org	21	18
	http://extremeriver.org	14	13
6) Overdose and suicide	http://www.a1b2c3.com	31	23
	http://www.ncbi.nlm.nih.gov	7	5
	http://www.everything2.com	5	5
7) Religion and suicide	http://www.religioustolerance.org	16	13
	http://www.believers.org	13	11
	http://en.wikipedia.org	7	5
8) Physician-assisted suicide	http://www.assistedsuicide.org	13	11
	http://www.religioustolerance.org	10	9
	http://www.amsa.org	5	5
	http://www.euthanasia.com	5	5
9) Discussion forum	http://www.xanga.com	14	2
	http://www.zenhex.com	13	12
	http://lifeflame.6.forumer.com	5	5
10) Families and friends of suicide/attempt	http://www.suicidememorialwall.com	9	4
	http://www.survivorsofsuicide.com	7	6
	http://www.parentsofsuicide.com	5	4
11) Afterlife	http://www.near-death.com	5	3
	http://samvak.tripod.com	3	1
	http://www.survivorsofsuicide.com	3	2

### Search Terms Used to Find Pro-suicide Websites

Among the 1314 accessed webpages, we could identify only two clicked-through websites that were considered pro-suicide. They are “http://www.suicidemethods.net” and “http://www.churchofeuthanasia.org”. The two websites not only provide information on methods of suicide but also portray a positive attitude towards the choice of using suicide as a way to ease pain. Table 3 shows the searches terms that were used to access the two websites (ie, “how to kill yourself”, “best way to commit suicide”, and “euthanasia”), the frequency of their usage, and the number of unique users for each search term. We found that the commonly used search terms included “pictures of murder by strangulation” and “photo of a severe burn” for “http://www.suicidemethods.net” and “committing suicide with a gas oven”, and “hairless goat” for “http://www.churchofeuthanasia.org”. It is noteworthy that 23 and 14 unique users visited “http://www.suicidemethods.net” and “http://www.churchofeuthanasia.org” respectively during the data collection period. It is important to view these numbers in context, especially given the fact that a single unique user contributed to the entire search query frequency of both “committing suicide with a gas oven” and “methods to commit suicide”, while the top two of search query frequency for “http://www.suicidemethods.net” were a result of only two unique users.

**Table 3:** Search terms used to access the two pro-suicide websites.

Website	Search term	Frequency	No. of unique users
http://www.churchofeuthanasia.org	committing suicide with a gas oven	27	1
	hairless goat	22	1
	how to kill yourself	18	3
	methods to commit suicide	11	1
	euthanasia	10	2
	best way to commit suicide	5	1
	butcher pig	4	1
	committing suicide	4	1
	how to die painlessly	3	1
	the church of euthanasia	1	1
	butchering the human carcass for human consumption	1	1
	dog sex kkh	1	1
http://www.suicidemethods.net	pictures of murder by strangulation	26	1
	photo of a severe burns	22	1
	pro choice suicide	7	1
	suicidal websites	6	1
	suicide murder pics	6	1
	suicide pics	6	1
	suicide methods	5	2
	asphyxia pics	4	1
	gory pictures	3	3
	gory photos	2	2
	homicide pictures	2	2
	pictures of suicides	2	2
	suicide	2	1
	bloody suicide jump pictures	1	1
	cut wrist pictures	1	1
	gory autopsy pictures	1	1
	hanging suicide	1	1
	shotgun wounds	1	1
	suicide attempt	1	1
	suicide and hell	1	1
	suicide photos	1	1

## Discussion

This is a retrospective observational study aimed to investigate the naturalistic web searching behavior on a search engine by online users looking for content using suicide-related keywords. Specifically, we examined users’ search terms with the keyword “suicide*”* and investigated the types of webpages they had accessed in the search results of AOL’s search engine, one of the major search engines in the United States during the data collection period. As such, the database used in this study might be dated, confined to an English-dominated search engine, and mainly limited to North America; nonetheless, this is the most comprehensive database of its kind that is freely available in the public domain for exploration. Due to online privacy developments, no other comparable publicly available datasets exist. Although using Google’s search trend tool, Google Trends, may provide some data on searching behaviors, comparisons would be difficult on many levels, including sampling ambiguity, lack of information on absolute search numbers, normalization of search results, lack of details on scaling of search results, and inability to access fine-grained searches.

Despite being a older dataset, especially in the context of the Internet where content and usage behavior is constantly changing, the findings still have relevance because, unlike prior studies that took a more computational perspective to suicide-related Internet searches, we used a naturalistic approach by looking at the users’ actual search behaviors. Rather than merely looking at what is available for our perusal on the Internet, which is subject to constant change, we examined the subsequent behavior with returned results, which seems relatively consistent even over time [29,30]. Furthermore, the detailed results of the search terms that we have been able to access through this dataset are not currently available anywhere since search trend tools are not able to drill down to this level of detail, and therefore, this may be the only glimpse into detailed search behavior of online users.

Utilizing this type of dataset, however, means that although users’ search result click-throughs were logged, we do not have a record of the search results webpages they could see and potentially access. Also, users’ behavior after accessing the webpages cannot be examined. Nevertheless, it is possible to identify search trends, patterns, and popular click paths that allow us to gain insight into users’ searching goals and intent as well as pages that had high visibility based on the keywords used in the search queries.

Presumably there are many existing websites that contain information about suicide, making the Internet’s role in suicides a point of concern. Our findings highlight three important findings on Internet users’ naturalistic web search behaviors that may challenge the underlying assumptions behind past studies suggesting that the Internet is intrinsically harmful because pro-suicide information can be found easily using suicide-related search terms. First, despite previous studies finding that pro-suicide and how-to-commit-suicide websites are easily accessible through search engines [15,21-23], our study showed that for AOL searches with suicide-related search terms, users generally accessed webpages in the search results that provided entertainment, scientific information, news, and resource information. About 10% of webpages accessed included information about specific means of suicide, or about 15% if overdose and suicide webpages are included. These preliminary findings could be interpreted as an indication of users’ search intent when they use suicide-related search terms. Second, only a very small proportion of users visited websites in the search results that were traditionally seen as potentially negative or harmful websites and among the clicked-through webpages, we could identify only two websites that may be considered as pro-suicide, showing detailed information of how to complete suicide and encouraging viewers to use suicide as a problem-solving strategy. Third, further investigation revealed that the majority of users did not use search terms that contained the keyword “suicide” to gain access to the two pro-suicide websites. More interestingly, according to the search terms that were used to access the pro-suicide websites, users may have been searching for gory images of unnatural deaths rather than for descriptive information on ways to commit suicide.

### The Internet and Suicide Prevention

In traditional media, newspapers, radio, and television were the major sources for information and their consumers were perceived as passive receivers of information. Information portrayed in traditional media was mainly contributed by professional journalists, and hence, regulation of that information was relatively straightforward. In terms of suicide information in traditional media, since information about suicide cases are presented and received passively by the masses, media reporting guidelines on suicide information developed for professionals has been considered as a core prevention strategy [31]. With the development of the Internet, we have stepped into the information age, which has revolutionized the ways we consume and produce information and transformed how we organize knowledge [32].

In the Web 2.0 era, the new media is interactive. People become proactive consumers and producers of information. Not only can people search for information and contribute, messages and information can also be widely and easily shared among friends via social media and networks. The ease and low cost of creating and copying content on the Internet contributed to its exponential growth and now, the amount of information that can be found on the Internet can be considered as almost infinite. In 2004, Google’s whole data storage was approximately five petabytes, which is the quantity of data a thousand times larger than the Library of Congress’s print collection [33]. According to a recent survey, there are 346,004,403 websites on the Internet as of June 2011 [34], and Google announced on July 2008 that it had processed over one trillion unique URLs [35], while Bing estimated that there were one trillion pages of content in 2009 [36]. Internet users, who can now also be contributors of information, do not have any obligations to follow reporting guidelines. Furthermore, content on the Internet can be easily copied, re-posted, cached, and reproduced in mirror-websites, and blocked websites can be bypassed easily through encrypted connections. A good example is the WikiLeaks site “http://en.wikipedia.org/wiki/WikiLeaks”, which had many mirror websites created when authorities tried to shut down and remove all the content from original website. The properties of this new medium make restricting access to particular websites difficult and impractical. Although the exact number of pro-suicide websites on the Internet is unknown, it is close to impossible to remove such websites from the Internet, regardless of whether they are easy or difficult to access.

### Actual Behavior and Intent of Suicide-Related Searches Online

Internet users use the Internet for various reasons. Segev and Ahituv conducted a cross-national analysis of popular search queries used in Google and Yahoo! over a 24-month period from January 2004 to December 2005 and found that there are cultural differences related to Internet usage [10]. They found that in many English-speaking and Western countries, the most popular searches were related to entertainment. According to the Pew Internet and American Life Project, information searches and email are the two major online activities among adult Internet users [37], and communication and entertainment are the most common ones among young people [38]. Accordingly, the “uses and gratifications theory” [39], a well-known theory in the field of media studies, states that the information people choose to access is largely dependent on their media consumption needs and desire. In other words, although negative information is easily accessible through new media, whether people access it largely depends on what they are looking for.

Our findings have provided a glimpse into the information consumption preferences of online users and corroborates prior studies on people’s Internet usage. Even for a domain-specific topic, suicide, our study generated consistent findings that most search engine users in the United States used suicide-related queries to search for websites that belong to the entertainment category.

Although pro-suicide websites and websites that contain detailed information on suicide methods can be accessed easily, the majority of search engine users, at least in the United States, did not access them using suicide-related queries. A number of keywords used to access pro-suicide webpages were instead related to violent or bloody pictures including “gory pictures”, “gory photos”, and “homicide pictures”. With these queries, we suspect that those who accessed the websites might not have an intent to die. Westerlund [40] has suggested that the fascination with pro-suicide content, particularly the morbid and violent descriptions may be a manifestation of a meaningful process in which the producer or consumer of this content is building an identity, acting out aggressive impulses, or even rebelling against the dominant culture. An interesting future study would be to explore the motivations behind why non-suicidal individuals access suicide-related information. The AOL data are very much based on a Western population and according to Segev and Ahituv’s findings [10], we do not know whether people in other countries behave similarly or differently. Recently developed free tools, like Google Trends and Google Insights for Search, may help to conduct a cross-national comparison study on this topic. With that information, however, we are still far from understanding how Internet users process information collected from the Internet and whether pro-suicide information leads to suicidal behaviors.

In-depth interviews with individuals with nearly lethal suicidal behavior may help to understand this phenomenon. In Biddle et al’s study [25], 22 individuals who survived nearly fatal suicide attempts were interviewed on the information sources that informed their choice of suicide method. They found that about 36% of the attempters’ choices of suicide method were found through the Internet. Interestingly, according to the transcripts cited in their paper, some attempters were “inspired” by online footage of Saddam Hussein’s execution or Wikipedia, which contains detailed information on suicide methods despite being a general information website. They wrote “the sites accessed by these respondents tended to be those containing professional information and resources (including online chemists), general knowledge sites (eg, Wikipedia), or news sites, including BBC news. Specific ‘suicide sites’ were accessed less frequently…” (p.705 of [25]). This information is essential to inform further development of suicide prevention work on the Internet.

In our study, we found that a small group of individuals intentionally searched for information about ways to complete suicide and an even smaller group of individuals were eager to find discussion forums using suicide-related keywords. A few individuals used queries like “suicide by overdose of XX” or “suicide by XX” to get access to webpages that provide information on prescribed drugs and suicide. In contrast, websites that include information on suicide means were accessed by larger numbers of unique users. One individual used a specific discussion forum address as a query (“xxsuicide’s xanga site” as shown in the table) to get access to that particular webpage 10 times.

There is a lack of research on suicide pacts arranged online, and so it is difficult to predict the major consequence of a large number of discussants getting access to this sort of discussion forum. On one hand, there have been some documented instances of completed suicide pacts in Japan [18], and on the other hand, there is evidence for the positive effects of joining an online community, like Facebook, which may enhance ones’ positive affective state and also increase ones’ social support [41].

### What Can Be Done to Minimize the Negative Impact of the Internet on Suicide?

Current efforts to reduce the potential negative impact of the Internet on suicide include remedial actions like removing and blocking entries of harmful websites by using Internet filters, constant monitoring of potential harmful websites by “web-based police officers” as implemented in Japan and Korea, and displaying helpline resources information while users key in suicide-related queries. These strategies are in place in an attempt to either shield suicidal and vulnerable individuals from suggestive material or abort their suicidal wishes by diverting their attention to help resources. There are several challenges that these strategies face. First, these strategies do not observe the way that information is created, found, and accessed online. Accordingly then, restricting or removing potentially harmful websites is an arduous if not impossible task. Furthermore, cyber regulations are extremely difficult to enforce since the Internet is not clearly under any jurisdiction, and identification of a physical user can be elusive.

Instead, it is important to further investigate the natural online behavior especially of vulnerable individuals and integrate that information with the way that search engines provide information. With the Internet inundated with information, the major search engines, ie, Google, Bing, and Yahoo!, have complex algorithms to return relevant search results to users. Consistent with the uses and gratification theory, relevance in a search engine is defined by variables such as user popularity, credible web domains, and high-quality content. As such, well-made and informative websites will tend to rank higher in search results and also be visited more often. Consequently, instead of the labor-intensive strategy of finding, sorting, and removing potentially harmful websites, more resources and effort should be spent on developing high-quality, informative, interactive, and user-friendly websites that maximize the likelihood of ranking highly in search results and therefore, being found and accessed.

Also, more recent studies find that the Internet provides more positive than negative effects on the vulnerable, especially in enhancing the social support of isolated individuals though social networking sites [11,42]. Mental health providers and researchers should make use of Information and Communication Technology (ICT) in strengthening their traditional practices in a fashion that could not be achieved in pre-digital times.

### Conclusion

Our research presented the naturalistic search behavior of search engine subscribers using any search terms with the keyword “suicide”. Currently we know that cybersuicide or Internet suicide pacts represent a small fraction of overall suicides. While those attempting suicide have searched for suicide-related information on the Internet before completing the act, we also know that the Internet can provide a unique platform to reach those individuals previously inaccessible, and web-based psychotherapies are producing promising results in helping depressed individuals, with the acknowledgment of the potential digital divide phenomenon. As such, it can be concluded that we should neither underestimate nor overestimate the potential negative impact of the Internet on suicide.

However, the threshold between offline and online activities is quickly receding and in the near future will soon disappear. It is uncertain how much of today’s searching, and even learning, behavior will remain unchanged. There are plenty of daily examples that show how much of our learning behavior has transformed. “*Siri” (*Speech Interpretation and Recognition Interface) is an intelligent personal assistant and knowledge navigator that functions as an application for Apple’s operating system and answers questions, makes recommendations, and performs actions by delegating requests to a set of web services to iPhone users. Google Images, a searching tool for images on the Internet, may help Google users to search for visual information using image or pictures. These are just a few examples. Suicide research and prevention strategies must keep up with the emerging trend and pace of ICT in order to prevent more unnecessary tragic deaths.
